# Current and future precision therapy approaches in the long QT syndrome

**DOI:** 10.1515/medgen-2025-2015

**Published:** 2025-07-17

**Authors:** Saranda Nimani, Miriam Barbieri, Marina Rieder, Katja E. Odening

**Affiliations:** University Hopital Bern and University of Bern Department of Cardiology and Department of Physiology Bühlplatz 5 3012 Bern Switzerland; University Hopital Bern and University of Bern Department of Cardiology and Department of Physiology Bühlplatz 5 3012 Bern Switzerland; University Hopital Bern and University of Bern Department of Cardiology and Department of Physiology Bühlplatz 5 3012 Bern Switzerland; University Hopital Bern and University of Bern Department of Cardiology and Department of Physiology Bühlplatz 5 3012 Bern Switzerland

**Keywords:** Gene therapy, Precision medicine, Cardiac channelopathies, Long QT syndrome, Sudden cardiac death

## Abstract

The long QT syndrome is a genetic arrhythmia disorder that predisposes patients to ventricular arrhythmias and sudden cardiac death. Pronounced genotype-specific differences in molecular mechanisms, arrhythmia triggers, and arrhythmogenic risk are well established, making the disease a prime candidate for precision medicine approaches in cardiology.

In this review, we first highlight the genetic basis of long QT syndrome, clinical genotype differences, and risk prediction approaches. In the second part, we discuss the current standard therapies applicable to all genotypes, as well as both established and emerging gene-specific precision therapy approaches.

## 1 Long QT syndrome – genetic basis, diagnosis and risk stratification

Congenital long QT syndrome (LQTS) is an inherited cardiac arrhythmia syndrome with an estimated prevalence of at least 1:2500 [1]. Despite being a rare disease, it is a major cause of sudden cardiac death (SCD) in the young (< 35 years) [2]. In LQTS, a dysfunction of cardiac ion channels leads to a delayed and dispersed myocardial repolarization, represented by a prolonged heart rate-corrected QT interval (QTc) on the electrocardiogram (ECG). This predisposes affected patients to potentially life-threatening ventricular arrhythmias, syncope, and SCD [3].

LQTS encompasses two distinct hereditary forms: the Romano-Ward syndrome and the Jervell and Lange-Nielsen syndrome (JLNS). The Romano-Ward syndrome follows an autosomal dominant inheritance pattern and presents with a broad phenotypic spectrum, ranging from completely asymptomatic individuals to patients developing sudden cardiac death. This clinical heterogeneity is among others influenced by genetic and/or hormonal modifiers [4, 5], resulting in significant phenotypic variability – even among individuals carrying the same genetic variant [6, 7]. As such, the clinical course cannot be reliably predicted based solely on the causative variant. In contrast, JLNS follows an autosomal recessive inheritance pattern and is associated with a more severe clinical course, characterized by marked QTc prolongation and concomitant congenital deafness [6].

To date, variants in 23 genes have been described in association with LQTS, although only 9 have sufficient evidence to support a causal role [6]. Genetic testing in LQTS index patients has a yield of about 75 % – with 90 % of genotype-positive cases harboring variants in one of the three “classical” LQTS genes – *KCNQ1,* *KCNH2* and *SCN5A* [3], the only genes for which precision therapy approaches have thus far been studied. The corresponding LQTS subtypes LQT1 (*KCNQ1*), LQT2 (*KCNH2*) and LQT3 (*SCN5A*) account for 40–55 %, 30–35 % and 5–10 % of all LQTS cases, with different molecular mechanisms leading to QTc prolongation. In LQT1 and LQT2 dominant negative loss-of-function (LOF) variants in *KCNQ1* (encoding the K^+^ channel K_v_7.1 conducting the slow delayed rectifier *I*_Ks_ current) or in *KCNH2* (encoding the K^+^ channel K_v_11.1 conducting the rapid delayed rectifier *I*_Kr _current), lead to prolonged repolarization. In contrast, LQT3 is caused by gain-of-function (GOF) variants in *SCN5A* (encoding the depolarizing Na_v_1.5 channels conducting *I*_Na_), which leads to a persisting late *I*_Na_ current [3].

The different molecular mechanisms lead to distinct clinical presentations and distinct arrhythmogenic triggers. LQT1 patients are at high risk of arrhythmias during exercise (such as swimming) or emotional stress, both of which are associated with a high sympathetic tone [8]. This is because the *I*_Ks_ current (that is defective in LQT1) is normally activated by adrenergic stimuli, resulting in QT shortening at increasing heart rates. The dysfunction of the *I*_Ks_ current leads to a pathognomonic exercise-induced QTc prolongation, thus, exercise stress testing can unmask concealed LQT1. LQT2 patients are at high risk of arrhythmias in situations of sudden startle (e. g., alarm clock ringing) [8]. In contrast, LQT3 patients are most prone to arrhythmic events at low heart rates, i. e., at rest or during sleep [8].

Risk stratification commonly takes into account sex, genotype and resting QTc [9]. In addition, we have previously demonstrated in a small cohort of LQTS patients that assessing post-exercise QTc – rather than resting QTc – considerably improves risk prediction in LQT1 patients [10], indicating gene-specific differences in risk assessment.

According to current guidelines, the clinical diagnosis of LQTS can be established if the QTc exceeds 480ms on repeated 12-lead-ECG, regardless of symptoms, or if the patient scores at least 3.5 points on the diagnostic “Schwartz score” (which takes into account QTc, T-wave morphology, arrhythmias/syncope, and a potential family history), or in the presence of a pathogenic or likely pathogenic LQTS variant [11]. Genetic testing is recommended for all index patients and for first-degree relatives when a pathogenic or likely pathogenic variant – rather than a variant of uncertain clinical significance – is identified in the proband. Genetic counselling is particularly important in the context of predictive testing for asymptomatic at risk family members [11].

All LQTS patients are advised to follow general recommendations to prevent life-threatening ventricular arrhythmias, such as avoiding QT-prolonging drugs, correcting electrolyte imbalances, and avoiding the mentioned gene-specific arrhythmia triggers [11].

**Figure 1:**
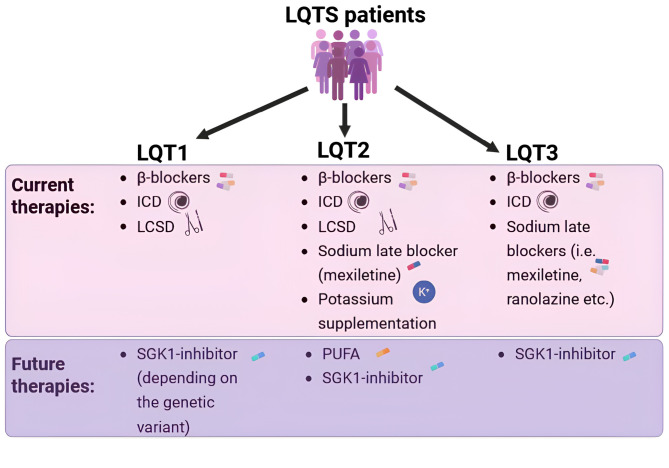
Current and novel precision therapy approaches in LQTS: Schematic representation of the current and novel precision therapeutic approaches in the long QT syndromes management. LCSD: Left cardiac sympathetic denervation; ICD: Implantable cardioverter-defibrillator; PUFA: polyunsaturated fatty acids; SGK1-inh: Serum and Glucocorticoid Kinase inhibitor.

β-blockers are the cornerstone treatment for LQTS, as they significantly reduce the incidence of arrhythmias (Figure 1) [12]. In general, non-selective β-blockers (such as propranolol and nadolol) are superior to cardio-selective β-blockers in preventing cardiac events in LQTS patients [13], due to their additional Na^+^ channel blocking effects, which cardio-selective β-blockers do not exhibit [13]. Therefore, current guidelines recommend the initiation of propranolol or nadolol as Class I recommendation in symptomatic patients [11]. However, side effects and medical contraindications to β-blockers (such as asthma) are relevant limiting factors in achieving optimal therapeutic dosage, and noncompliance is relatively common [14]. Overall, up to 32 % of all symptomatic patients may experience another arrhythmic event despite therapy [12].

In LQT3, the efficacy of β-blockers is less pronounced compared to LQT1 and LQT2, as β-blocker-induced bradycardia may aggravate arrhythmias in this LQTS subtype [8]. However, the late *I*_Na_ blocking agent Mexiletine has been shown to effectively prevent arrhythmias in LQT3 patients [15], as discussed in the following chapter on gene-specific therapies.

In special situations, such as recurrent arrhythmias associated with β-blocker intolerance, left cardiac sympathetic denervation (LCSD) is a possible therapeutic option [11].

Additionally, the implantation of an implantable cardioverter-defibrillator (ICD) is a Class I recommendation for all patients who remain symptomatic despite β-blocker (or mexiletine in LQT3) therapy, as well as for all patients with resuscitated cardiac arrest [11].

However, as LQTS often manifests at a young age, the long-term complications of ICD therapy such as material exhaustion or recurrent battery replacement, must be given special consideration, highlighting the need for more efficient, gene-specific treatments [16].

## 2 Gene-specific therapy approaches in the long QT syndrome

### 2.1 Current approach: Mexiletine therapy in LQT3 and LQT2

The use of mexiletine, a sodium channel blocker, for patients diagnosed with LQT3 was first proposed in 1995, making it the first precision, gene-specific therapy for LQTS. Mexiletine shortens the pathologically prolonged QTc by inhibiting the abnormally enhanced late *I*_Na_ [17]. Notably, Schwartz et al. demonstrated that mexiletine shortened the QTc by 90ms in six patients with LQT3 during an acute oral drug test [17]. These findings were further supported by a cohort study conducted by Mazzanti et al. with 34 patients, which confirmed the QTc shortening efficacy and demonstrated that it was particularly effective in patients at highest risk, especially those with a prolonged QTc > 500ms. Moreover, mexiletine reduced the risk of arrhythmias [18]. These significant results have led to a Class I treatment recommendation for patients with LQT3 [11], making mexiletine an essential component of therapy for these patients (Figure 1).

It is logical that mexiletine, as a late *I*_Na_ blocker, is effective in treating LQT3 with a gain of function in late *I*_Na_. However, several experimental studies conducted in animal models, tissues, and human induced pluripotent stem cell-derived cardiomyocytes (hiPSC-CMs) suggest that late *I*_Na_ blockade may also effectively shorten the QTc and prevent arrhythmic events in LQT2, where late *I*_Na_ is not primarily affected by the underlying causative mutation. For instance, mexiletine was shown to shorten the action potential duration (APD) and reduce proarrhythmic APD dispersion in drug-induced LQT2 canine wedge preparations [19]. Additionally, it was found to shorten cardiac repolarization in hiPSC-CMs derived from LQT2 patients and in transgenic LQT2 rabbit models, where late *I*_Na_ is indeed secondarily enhanced, compared to wild-type (WT) rabbits [20]. These promising experimental findings are further substantiated by clinical studies. A retrospective analysis of a small cohort of 12 LQT2 patients in 2019 revealed that mexiletine shortened QTc by an average of 65ms [21]. Recently, Crotti et al. [20] conducted a comprehensive investigation into mexiletine’s efficacy in treating LQT2, showing significant reductions and prevention of arrhythmia recurrences in a large cohort of 96 LQT2 patients. Notably, the response to mexiletine was largely dependent on baseline QTc, with the greatest benefit observed in patients with a baseline QTc greater than 500ms, in whom a substantial antiarrhythmic efficacy was also observed.

### 2.2 Future approaches

#### 2.2.1 PUFA as a novel potential treatment in LQT2

Among treatment strategies aimed at restoring the physiological QTc, a key focus is on addressing the underlying mechanistic causes of LQT1 and LQT2, e. g., the defective *I*_Ks_ and *I*_Kr_ [3]. Many groups have focused on the development of activators for *I*_Ks_ currents, but to date no clinically approved *I*_Ks_ activator exists. One promising area of investigation involves polyunsaturated fatty acids (PUFAs), which have been identified as modulators of voltage-gated ion channels, capable of activating I_Ks_ currents [22, 23]. Studies by Skarsfeld et al. assessed the potential of natural and modified PUFAs to shorten the QTc in healthy and drug-induced LQTS guinea pig hearts in *ex vivo* whole heart and *in vivo* experiments. They found that DHA (docosahexaenoic acid), DHA-GLY (docosahexaenoic acid-glycine), Lin-GLY (linoleoyl glycine) all significantly shortened the QTc due to their pronounced *I*_Ks_ activating effect [24]. However, these *I*_Ks_ activators only work in normal *I*_Ks_ conducting channels and not in channels harbouring mutations in their α- or β-subunit. Taking these experiments further, Castiglione et al. investigated the potentially beneficial effects of DHA on QTc and APD in transgenic rabbit models for LQTS (LQT1, LQT2, LQT2–5, and LQT5), examining potential gene-specific effects. They demonstrated that DHA exerts a beneficial shortening and normalizing effect on QTc and APD only in LQT2 rabbits, which carry a defective *I*_Kr_ but have a normal functioning *I*_Ks_, with no effect observed in LQT1, LQT2–5, or LQT5 – all of which have defective *I*_Ks_ channel function due to mutations in the α-subunit of *KCNQ1* or the β-subunit of *KCNE1*. Thus, they hypothesized that DHA could represent a new gene-specific therapeutic option for LQT2 or other LQTS subtypes with intact α- and β-subunits of I_Ks_ (Figure 1) [25].

#### 2.2.2 SGK1-inhibition as a novel potential treatment in LQT1, LQT2 and LQT3

Beyond approaches targeting mutated channels (such as mexiletine in LQT3 or PUFAs in LQT2), novel strategies are now focusing on preventing pro-arrhythmic events that result from APD prolongation (due to the underlying mutations), resulting in disruption of normal sodium and calcium homeostasis [26]. These methods are currently being investigated as potential therapeutic targets for various LQTS subtypes, particularly in patients at high risk of lethal ventricular arrhythmias or those in whom conventional treatments (such as β-blockers) have failed.

Several studies have focused on the serum and glucocorticoid kinase-1 (SGK1), an important regulator of Na_v_1.5-mediated *I*_Na_ in the heart [27, 28]. SGK1 overactivation increases late *I*_Na_, prolongs APD, and induces ventricular arrhythmias, recapitulating the LQTS phenotype [28]. A proof of concept for SGK1-inhibition as a therapy for LQT3 was provided by Bezzerides et al, who demonstrated an APD-shortening effect in patient-specific LQT3 hiPSC-CMs by SGK1-inhibitors [29]. Given the effectiveness of SGK1 inhibition in LQT3 models, additional studies have explored its impact on LQT1 and LQT2 models, since several findings suggest the (secondary) involvement of enhanced late *I*_Na_ in these genotypes as well [20, 30]. Gianetti, Barbieri et al. [31] investigated pharmacological SGK1-inhibition in a variety of *in vitro* and *ex vivo* models of LQT1 and LQT2, including hiPSC-CMs and rabbit models. They found that SGK1-inhibition shortened the APD by 20–30 % across different model systems, species (human and rabbit), and *KCNH2* pathogenic variants, suggesting the generalizability of this effect. Conversely, the effects of SGK1-inhibition in LQT1 appeared more complex, showing variable responses in three different *KCNQ1* variants, indicating a potential variant-specific effect (Figure 1). Additionally, another SGK1-inhibitor was tested by Kim et al. [32] revealing significant APD shortening in patient-specific hiPSC-CM models of all three genotypes, LQT3, LQT1 and LQT2. While this study indicated a more consistent benefit in LQT1, it only investigated one pathogenic *KCNQ1* variant, leaving uncertainty about whether the efficacy is due to the different molecules tested or the specific properties of the *KCNQ1* variant studied.

## 3 Gene therapy approaches for LQTS

Despite substantial progress in the treatment of LQTS, available treatment options do not address the underlying genetic substrate of the disease [33]. This has led to a significant interest in exploring novel precision gene therapies for LQTS.

While the field of gene therapy has advanced at an extraordinary rate, its clinical application in cardiac diseases has somewhat lagged behind. In the recent years, however, many challenges associated with cardiac gene therapy have been overcome, setting the ground for several ongoing clinical trials for monogenic cardiac diseases [34].

**Figure 2:**
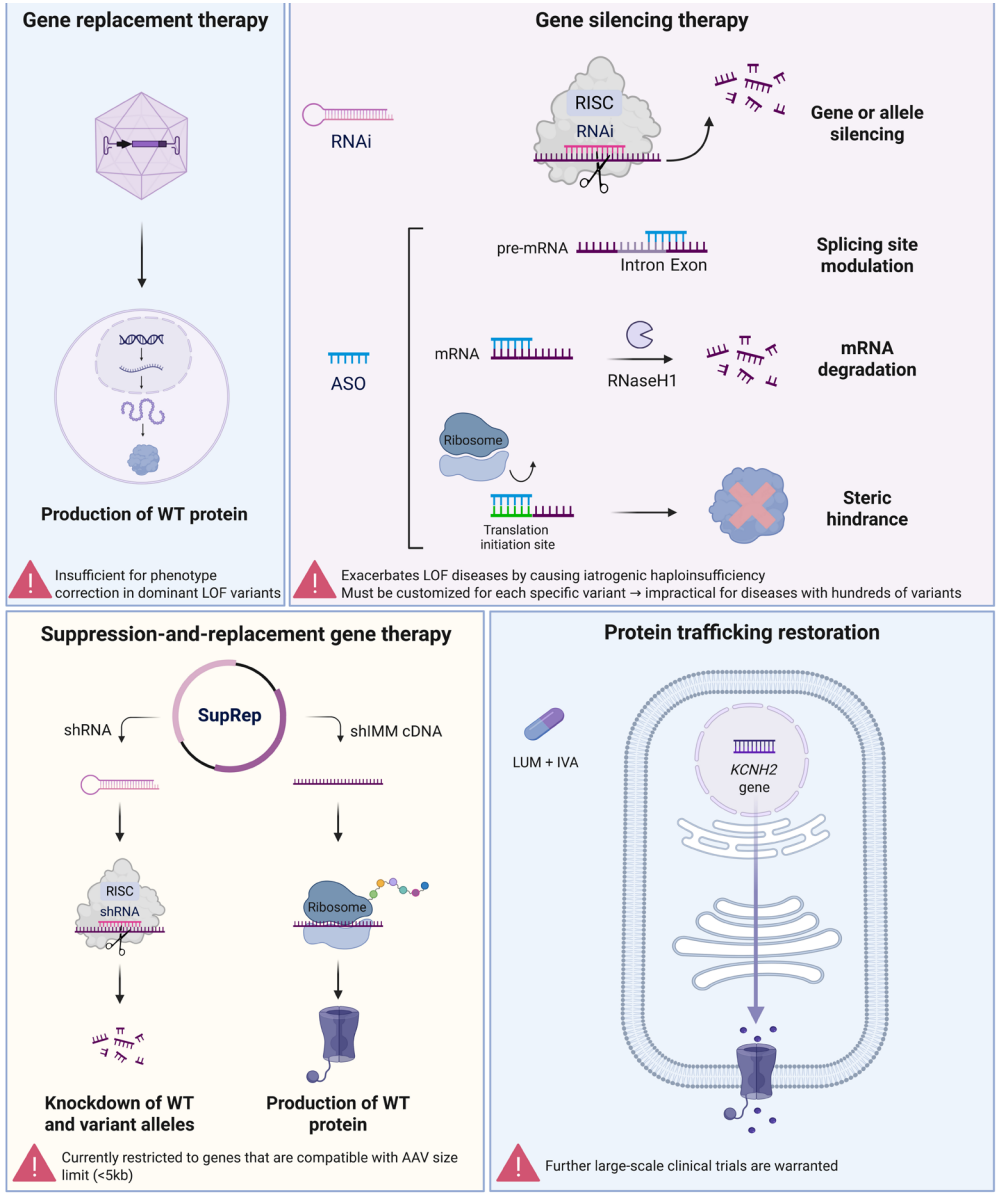
Future gene-specific therapeutic approaches for LQTS. A schematic overview of the key gene therapy strategies being explored for LQTS, including gene replacement therapy, gene silencing therapy, and suppression-and-replacement gene therapy. Note that genome editing is not presented here. Moreover, illustrated in the lower right panel is the protein trafficking restoration strategy using LUM + IVA for LQT2. mRNA: messenger RNA; pre-mRNA: precursor messenger RNA; RNAi: RNA interference; RISC: RNA-induced silencing complex; ASO: Antisense oligonucleotide; shRNA: short hairpin RNA; shIMM cDNA: shRNA-immune cDNA; LUM: Lumacaftor; IVA: Ivacaftor. Figure created with BioRender.com

Overall, the main gene therapy strategies include gene replacement therapy (GRT), gene silencing therapy (GST), genome editing (GE), and hybrid suppression-and-replacement (SupRep) gene therapy (Figure 2) [35].

### 3.1 Gene replacement therapy

GRT involves the introduction of a healthy, wild-type gene into target cells to replace a reduced or defective gene. GRT is typically utilized in the setting of LOF variants leading to haploinsufficiency [33, 35]. The first AAV-based GRT phase 1 clinical trial for cardiomyopathy has shown promising initial results for Danon disease – caused by *LAMP2* variants. A phase 2 clinical trial is currently underway [36]. In addition, similar AAV-based GRT approaches are being pursued for other cardiac diseases, such as *MYBPC3*-mediated HCM [37] and *PKP2*-mediated ARVC [38, 39]. Despite these successful applications in the cardiac field, a GRT approach is insufficient in addressing diseases caused by dominant-negative LOF variants such as LQT1 and LQT2, as the mutated gene acts as a ‘poison peptide’ that interferes with the WT product. This interaction reduces the number of functional WT tetramers, rendering GRT suboptimal for phenotype correction.

### 3.2 Gene silencing therapy

GST involves silencing of a disease-causative gene or allele, using strategies such as anti-sense oligonucleotide (ASO) or RNA interference (RNAi). GST is particularly useful for treating diseases caused by dominant-negative LOF variants as well as GOF variants. Encouraging preclinical results of GST approaches have been reported in experimental models of *MYH6*-mediated HCM [40], *MYL2*-mediated restrictive cardiomyopathy (RCM) [41], and *RYR2*-mediated CPVT2 [42]. Additionally, allele-specific RNAi approaches have been successfully applied to target specific variants in LQT1 and LQT2 [43, 44]. However, GST does not come without limitations. For one, GST must be customized for each specific disease-causing variant, which is impractical for diseases with hundreds of unique variants – such as LQT1, with over 490 reported pathogenic or likely pathogenic variants, and LQT2, with over 590 reported pathogenic or likely pathogenic variants in ClinVar (https://www.ncbi.nlm.nih.gov/clinvar/). Similarly, genome editing approaches face the same impracticality in LQTS, as each variant would require a uniquely tailored approach. Moreover, in the case of dominant-negative LOF variants, silencing the variant allele may not be sufficient; it must be complemented with replacement of the healthy, WT copy, to correct the disease phenotype beyond haploinsufficiency [35]. Thus, while GST offers promising therapeutic potential, its applicability to diseases like LQTS remains limited.

To overcome this challenge, Dotzler et al. developed a hybrid suppression-and-replacement (SupRep) gene therapy approach for LQT1 and LQT2 [45, 46].

### 3.3 Suppression-and-replacement gene therapy

SupRep gene therapy combines a custom-designed short hairpin RNA (shRNA) with its corresponding shRNA-immune (shIMM) cDNA within a single vector. The shRNA (suppression component) is designed to target a portion of the *KCNQ1* or *KCNH2* gene, devoid of common pathogenic variants, leading to knockdown of both variant- and WT-allele. This expands the applicability of the SupRep approach to all LQTS patients, regardless of their specific disease-causing variant. The shIMM cDNA then utilizes codon redundancy to evade shRNA suppression, while preserving the WT amino acid sequence, thus increasing WT protein expression [45, 46]. Indeed, in hiPSC-CMs from LQT1 and LQT2 patients, the *KCNQ1*- and *KCNH2*-SupRep therapies effectively shortened the pathologically prolonged APD [45, 46]. Additionally, we recently demonstrated the therapeutic efficacy of the SupRep approach in rescuing the diseased phenotype in a transgenic LQT1 rabbit model, both at rest and during adrenergic stress [47].

## 4 Restoring protein trafficking in LQTS

One of the main mechanisms underlying *I*_Kr_ impairment in LQT2 involves mutations associated with defective intracellular trafficking [48]. This has sparked considerable interest in developing novel approaches to correct trafficking defects [48]. The most promising therapeutic agent identified in this category so far is Lumacaftor (LUM), originally developed and validated for the treatment of cystic fibrosis [49]. Mehta et al. first reported LUM’s effectiveness in restoring *KCNH2/I*_Kr_ trafficking in hiPSC-CMs from LQT2 patients carrying trafficking variants, but not in those with biophysical alterations of the channel/current, highlighting its specificity for trafficking-related defects [50]. What makes LUM particularly appealing, is its already established clinical use and well-characterized safety profile [50]. To validate these *in vitro* findings, Schwartz et al. tested the LUM and Ivacaftor (IVA) combination – an established cystic fibrosis therapy – in the same LQT2 patients whose hiPSC-CMs had responded to LUM and observed a ~30ms QTc shortening [51]. While these findings are very encouraging, larger clinical trials are needed to confirm LUM’s therapeutic potential for LQT2.

## 5 Summary & outlook

Congenital LQTS is a common inherited arrhythmia syndrome that significantly increases the risk of SCD, particularly in young individuals [1, 2]. LQTS is characterized by a prolonged cardiac repolarization, represented by a prolonged QTc on ECG. The main LQTS subtypes – LQT1, LQT2, and LQT3 – are caused by mutations in the *KCNQ1*, *KCNH2*, and *SCN5A* genes, respectively, each with distinct gene-specific triggers and clinical presentations [3].

Despite substantial clinical progress, current treatment approaches for LQTS are mainly symptom-oriented and do not address the underlying molecular mechanism of the disease [33]. However, the outlook for LQTS treatment is promising, with advancements in gene-specific therapies and mechanism-based approaches. Mexiletine is a key gene-specific precision therapy for LQT3 [11] and has also shown potential for LQT2 [20]. The exploration of PUFAs [23] and SGK1-inhibition [29–32] might provide new therapeutic options for various LQTS subtypes. Gene therapy, particularly the hybrid SupRep approach, directly targets the genetic substrate of LQTS, with encouraging APD/QT normalizing results observed in hiPSC-CMs from LQT1 and LQT2 patients [45, 46], and in a transgenic LQT1 rabbit model [47]. Finally, LUM, a drug that restores protein trafficking in LQT2, represents a targeted therapeutic strategy that may improve outcomes, with promising preliminary results in both hiPSC-CMs and LQT2 patients [48, 50, 51]. However, larger clinical trials and further refinement of these therapies are needed to confirm their efficacy and safety. Overall, the field is evolving rapidly, with precision medicine paving the way for more effective treatment modalities for LQTS.
